# How the ovules get enclosed in magnoliaceous carpels

**DOI:** 10.1371/journal.pone.0174955

**Published:** 2017-04-21

**Authors:** Xin Zhang, Wenzhe Liu, Xin Wang

**Affiliations:** 1 College of Forestry, Northwest A&F University, Yangling, China; 2 College of Life Sciences, Northwest University, Xi’an, China; 3 State Key Laboratory of Palaeobiology and Stratigraphy, Nanjing Institute of Geology and Palaeontology, CAS, Nanjing, China; Wuhan University, CHINA

## Abstract

Angiosperms distinguish themselves from gymnosperms by their ovules that are enclosed before pollination. However, how the ovules get enclosed in angiosperms remains a mystery, especially for Magnoliaceae. The only key to this mystery is finding a series of carpels transitional from fully closed with enclosed ovules to open with naked ovules. We use routine paraffin section technology, LM, SEM to document the morphology and anatomy of carpel variation in *Michelia figo* (Magnoliaceae). A series of carpel variations within a single flower of *Michelia figo* (Magnoliaceae) are documented, in which the ovules are exposed in atypical carpels. These atypical and typical carpels for the first time demonstrate clearly how the naked ovule get enclosed. Each atypical carpel, with naked ovules, clearly comprises two parts, namely, subtending foliar part and branches bearing ovules, suggesting that a typical carpel is actually an end-product of the fusion between the ovuliferous branches and subtending foliar parts. The only difference among these carpels is the extent of fusion between these two parts. This generalization is in full agreement with the molecular genetic studies on angiosperm flowers.

## Introduction

The crucial step in the evolution leading to angiosperms is how the formerly naked ovules become enclosed. Although the APG III system has re-shaped the understanding of angiosperms [1], no sound solution is available for the origin of carpels [2]. According to the widely accepted Euanthium Theory, ovules in the ancestors of angiosperms are borne along the margins of a foliar part (so-called “megasporophyll”) and they become enclosed through longitudinal folding of such “megasporophyll”[3]. Ovules arrangement along ventral the carpel margins in the Magnoliaceae [4], the assumed uniform anatomy of magnoliaceous flowers carpels [5], the assumed conduplicate carpels and marginal placentation seen in the Early Cretaceous *Archaefructus*[6] all seem to favor this hypothesis. At the same time, this hypothesis is facing challenges from various works. For example, studies on the gynoecium of *Arabidopsis thanliana* indicates that the ovules can develop without carpel wall [7]; a so-called carpel is found derived from two primordia controlled by two sets of genes, and is supplied by two different and independent vascular systems (one for ovarian wall and one for the placenta) [8–11]; an ovule primordium demonstrates a juxtaposition of expression of REV and STM, similar to a shoot apex [12]; and carpels of *Archaefructus* are not conduplicate and do not have marginal placentation [13]. Despite all, magnoliaceous carpels remain especially revealing in term of the nature of “carpels”. Here we document the carpel variations in *Michelia figo* (Magnoliaceae) to shed some light on this question. The female units in the same flower of *Michelia* demonstrate two extremely different (atypical and typical) organizations that are bridged by a transitional series. The ovules in atypical female units are exposed and borne on two branches (placenta) independent of the subtending foliar parts, while the ovules in typical female units appear enclosed and borne along the margins of the foliar parts. The occurrence of these two extreme carpel types in a single flower suggests that ovules are oringinally borne on branches that fuse with the margins of the subtending foliar parts in typical magnoliaceous carpels.

## Materials and methods

### Material collection and preparation

The materials of the atypical female units were collected from living plants of *Michelia figo* cultivated in the Botanical Garden of the Ruhr University Bochum (Germany) during the period ranging from 2011 to 2013. The materials were preserved and dissected in 70% ethyl alcohol. Selected features were observed using a Zeiss Stemi SV11 stereomicroscope and photographed with a KEYENCE VHX-500F digital microscope. Then the material was fixed in FAA (formalin: acetic acid: ethyl alcohol 70% = 5: 5: 90) and kept in the fixative under moderate vacuum for at least 2 days. Afterward, the FAA was replaced by 70% ethyl alcohol for further storage. Fruits of *Michelia figo* were collected from a plant cultivated in the Nanjing, China in 2015. Paraffin sections were prepared from these materials according to the routine method [14]. Part of the paraffin sections were stained with Safranin O and Fast Green. Remove the wax from the sections with 2 times 15 min wash in 100% Xylene. Change the sections into a mixed solution of 100% ETOH(50%) and 100% Xylene(50%) for 5 min. Then bring through a graded ETOH series from: 100% ETOH—5 min, 100% ETOH—5 min, 95% ETOH—2 min, 85% ETOH—2 min. Stain more than 12 hrs in the 1% w/v Safranin O solution (75% ETOH). Normally, the sections stay in the Safranin O solution over night. Next morning, changed the sections in 85% ETOH for 5 min. Counter stain for just 10 to 15 seconds in Fast Green 0.05% w/v(95% ETOH). Test one slide at a time to prevent over-staining by the fast green. Use a minimal amount of time in stain. Change the sections into a mixed solution of 100% ETOH (50%) and 100% Xylene (50%) for 5 min. Clear in 100% xylene two times washes of 5–10 minutes each. Mount coverslip with Permount. Critically observed and photographed using a Nikon Eclipse 50i microscope with a Nikon DS-Fil digital camera. The other paraffin sections were stained with Aniline Blue, observed and photographed after excitation at 365 nm with a Leica DML epifluorescence microscope with a Leica DC300F camera.

### Scanning electron microscopy (SEM)

The specimens of interest were transferred from ethyl alcohol to dimethoxymethane and stored at 4°C for at least 48 hours. Dimethoxymethane chemically dehydrates the plant tissue and serves as intermedium in the critical point drying process [15]. Critical point drying was performed using a CPD 030 (BALZERS). Depending on size and structure of the material, the dried tissue was mounted on aluminum stubs either with conductive pads (Leit Tabs, PLANO) or conductive carbon cements (Leit-C, PLANO) and then stored in a desiccator with silica gel.

The samples were sputter-coated with gold for 200–400 s at 42–43 mA (BAL-TEC SCD 050). Scanning electron microscopy was performed with a DSM 950 (ZEISS). For documentation, a digital image processing system (DIPS 2.2, POINT ELECTRONIC) was used, which allowed the storage in the Tiff-format (2000 × 2000 pixels). The size was adapted to the plate format using Adobe Photoshop; the contrast in some images was enhanced, other image processing was not performed.

## Results and discussion

**Fig 1:**
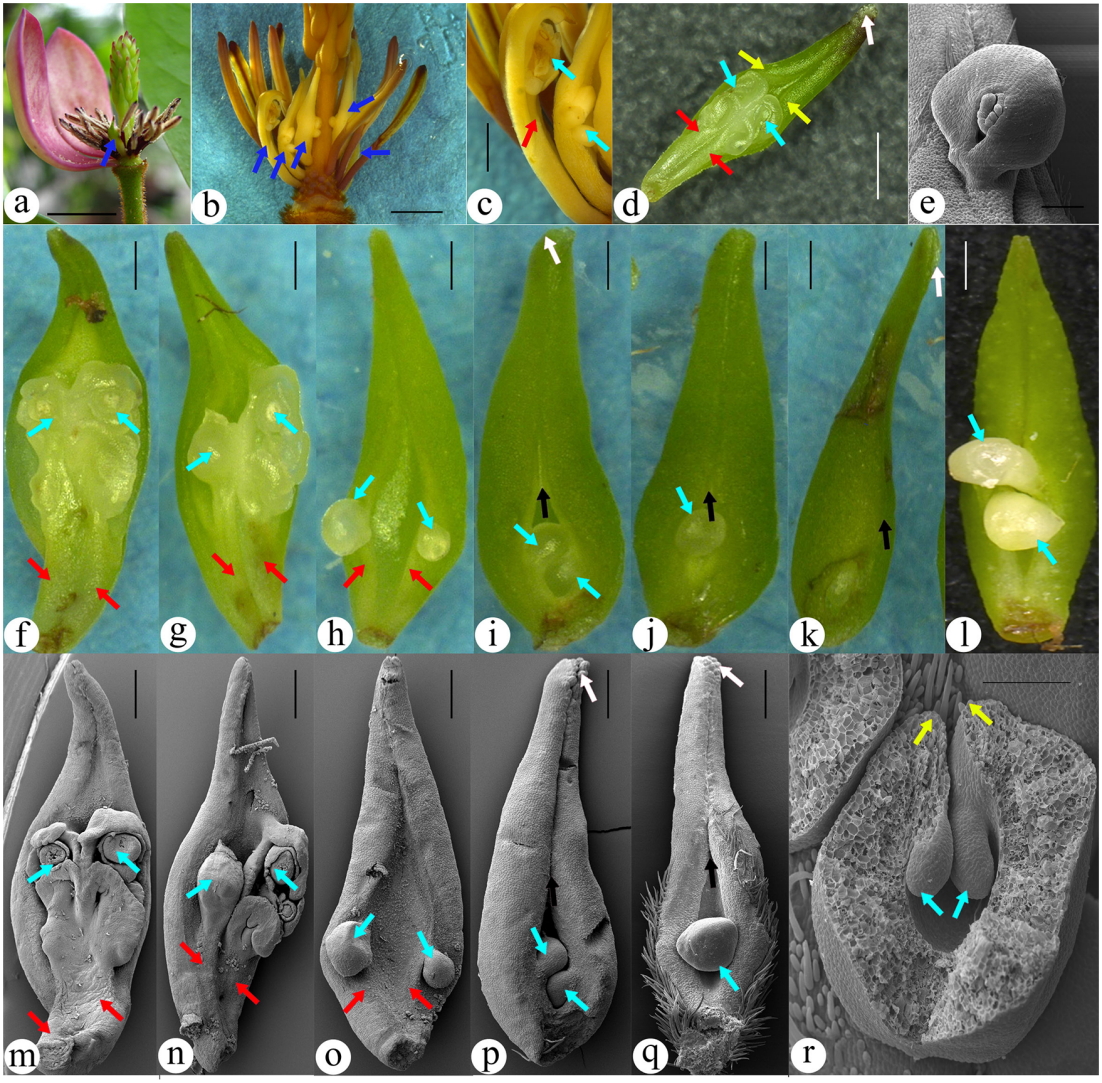
Configuration of atypical female units that are transitional to the typical one. **A-K are stereomicrography, L-S are SEM**. (A) A post-anthetic flower with an atypical female unit (arrow) situated between the male and female sections. The tepals are removed to show inner flower parts. Bar = 10 mm. (B) A flower with several atypical female units (arrows) between the male and female sections. Bar = 10 mm. (C) Detailed view of the atypical female units shown in Fig. 1B. Note the exposed ovules and that, in at least one female unit, the ovuliferous branch (placenta) is obviously separated (arrow) from the subtending foliar part. Bar = 1 mm. (D) Adaxial view of an atypical female unit comprising a subtending foliar part and a placenta in its axil. Papillae (blue arrow) and enrolling margins (white arrow) are seen on the distal of the foliar part. The placenta comprises two slightly fused branches (red arrows), each of which terminates in an ovule (yellow arrow). Bar = 1 mm. (E) One of the ovules in Fig. 1m that is attached to the placenta (red arrow) isolated from the subtending foliar part (black arrow). Bar = 0.5 mm. (F-Q) A serial pairs of LM and SEM images showing female units transitional from atypical to typical configuration. The spatial relationship between the ovules and subtending foliar parts changing from isolated gradually into increasingly fused, and the presence of ovuliferous branch is increasingly hard to see. Figs. 1f-j and 1m-q are from a single flower. Bar = 0.5 mm. (F, M) Anatypical female unit with a configuration similar to that in Fig. 1D. Note the barely fused branches (red arrows, placenta) terminating in ovules (yellow arrows). One of the ovule is shown in detail in Fig. 1e. (G, N) Ovules (yellow arrows) on the tips of branches (placenta, red arrows) subtended by a foliar part. (H, O) Two ovules (yellow arrows) appearing borne on the margins of the foliar part due to the fusion between the two branches (of placenta, red arrows) and foliar part margins. The foliar part has its margins (white arrow) enrolled in the distal portion. (I, P) Further enrolling of the foliar part giving rise to an obvious ventral suture (white arrow). Note the ovules (yellow arrows) are more enclosed than in Fig 2g and 2n. (J) Almost completely closed female unit with obvious ventral suture (white arrow) and only one ovule (yellow arrow) visible. (K) A completely closed female unit. Its ovule (yellow arrow) is fully enclosed and visible only when the female unit is cut at the bottom. (L) A closed female unit that fails to enclose its ovules. (Q) Almost completely closed female unit with obvious ventral suture (white arrow) and only one ovule (yellow arrow) visible. (R) A cross-cut typical female unit showing the ovules (yellow arrows) fused to the margins (white arrows) of the foliar part. Only this image was slightly horizontally squashed to fit into the space available. Bar = 0.2 mm.

**Fig 2:**
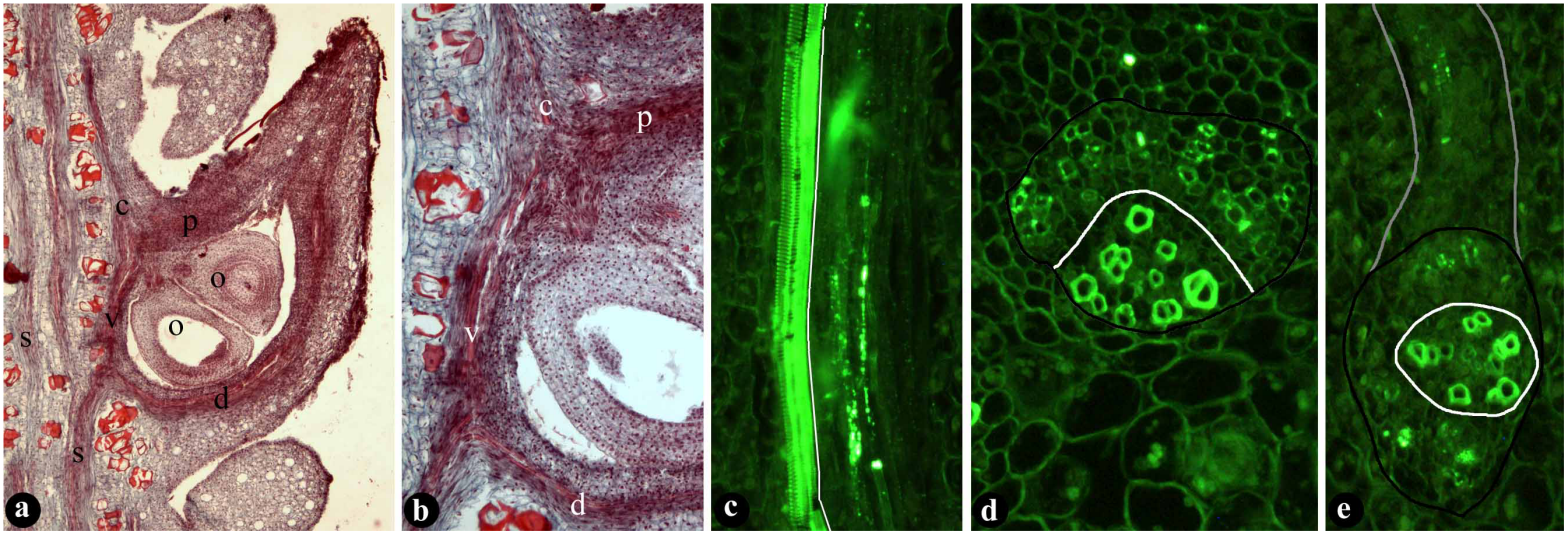
Anatomy of typical fruits showing vascular bundles in the fruit wall and placenta. (A) Longitudinal radial section of a fruit showing dorsal (d) and ventral (v) bundles, and placenta bundle (p) supplying the ovules (o). Bar = 1 mm. (B) Detailed view enlarged from Fig. 2a, showing dorsal bundle (d), ventral bundle (v), placenta bundle (p) supplying the ovules (arrows). Bar = 0.1 mm. (C) Longitudinal section of a collateral dorsal bundle in the fruit wall, showing adaxial xylem (to the left of white line) and abaxial phloem (to the right of white line). Bar = 50 μm. (D) Cross view of a collateral stellar bundle (black line) with adaxial xylem (below white line) and abaxial phloem (above white line). Bar = 20 μm. (E) Cross view of an amphicribral placenta bundle (black line) with xylem (within in the white line) surrounded by phloem (between the white and black lines). The bundles is extended (gray line) above to an ovule. Bar = 20 μm.

Both typical and atypical female units were observed in the gynoecium of *Michelia figo*, and all these female units can constitute a seamless transitional series (Fig 1d and 1f–1r). Some of these atypical and typical female units occur in a single flower (Fig 1f–1j and 1m–1q). The common feature of the atypical female units is that their ovules are naked and borne on branches (placenta) (Fig 1b–1i and 1m–1q), and each of female units comprises a placenta and a subtending foliar part (Fig 1c, 1d, 1f and 1m–1r). The placenta is clearly separated from the subtending foliar part, and the ovules are on the termini of ovuliferous branches, not along the margins of the subtending foliar parts (Fig 1c, 1d, 1f, 1m and 1n). As the adnation between the placenta and subtending foliar part proceeds, two ovuliferous branches separate from each other and each independently fuses with a margin of the subtending foliar part, resulting in a marginal-appearing placentation (Fig 1h, 1i, 1o and 1p). As the foliar parts enrolls, a ventral suture appears in the adaxial-distal first and extends proximally, while the ovules become increasingly enclosed (Fig 1h–1j and 1o–1q). In typical female units, the foliar parts are completely closed, and the ovules are fully enclosed and appear attached to the margins of the foliar parts (Fig 1k and 1r). Sometimes the foliar parts may have completed its closure but failed to enclose the ovules (Fig 1l). Anatomically, the dorsal and ventral vascular bundles of the fruits are derived from the same vascular bundle, and they all are collateral and amphicribral, respectively, in in the fruits of *Michelia figo* (Fig 2a–2e). The ovules are supplied by amphicribral placenta bundles (Fig 2a, 2b and 2e).

The observation of this phenomenon was carried out from 2011 to 2013. Every year, the phenomenon can be found. Twenty two samples were phoned in total during these three years.

The etymology of “angiosperm” *per se* implies that exposed ovules are not expected for angiosperms. Generally say, angiosperms are distinguished from their gymnosperm peers by their ovules that are enclosed before pollination [16, 17]. The ovules in the typical female units of *Michelia* are enclosed (Fig 1k and 1r), as expected. If exposed ovules of *Michelia* in Fig 1c–1j and 1l–1q were taken alone, this single feature alone would appear sufficient to justify a gymnospermous affinity for *Michelia*. Although ovules physically exposed to the external have been seen in other angiosperms (for example, Resedaceae) [4, 18], to our knowledge, naked and enclosed ovules have not been seen together in an individual plant, not mention in a single flower. Therefore the occurrence of both naked and enclosed ovules in a single flower of *Michelia* (Fig 1c vs 1f–1k and 1m–1p) provides a unique opportunity that opens a window allowing us to reveal the evolution of gynoecium in magnoliaceous plants.

As expected for the Magnoliaceae, the ovules in typical female units may be interpreted as borne along the margins of the foliar parts (Fig 1r), as expected by Arber and Parkin (1907). Similar situation is also seen in some atypical female units, *e*.*g*. the ovules in Fig 1h, 1i, 1l, 1o and 1p appear borne along the margins of the foliar parts (ovarian wall). However, the ovules in atypical female units tell a completely different story. In these female units, the ovules are borne on two branches and independent of the subtending foliar parts (Fig 1c, 1d, 1f–1g, 1m and 1n). This observation is in line with the shoot-nature of placenta suggested by studies on function genes, in which placenta is regarded as an ovule-bearing branch recruited onto the margins of subtending foliar part [12]. Actually, certain genes are restricted to placenta and ovules and never expressed in ovarian wall [12, 19]. The placenta and ovarian walls of *Magnolia* develop independently and are supplied by vascular bundles with different organizations in *Magnolia* [11]. Comprehensive analysis of interdisciplinary evidence indicates that the female units in angiosperms comprise placenta (ovuliferous branches) and placenta-enclosing foliar parts [17]. So both morphological observations (including the present study) and function gene studies agree each other on the shoot-nature of placenta in magnoliaceous carpels.

The marginal-appearing positions of ovules (Fig 1h–1j, 1o and 1p) may be interpreted as a result of the fusion between placenta and foliar part. The independence of placenta from the foliar parts is clearly seen in Fig 1c, 1d, 1g, 1m and 1n, and also clearly favored by the different organizations of vascular bundles in the placenta and foliar parts (Fig 2a–2e). The dorsal bundle in the female units is collateral (with adaxial xylem and abaxial phloem) (Fig 2a–2d). Similar bundles have been seen in leaves of seed plants [4], implying the foliar nature of ovarian wall. In contrast, the vascular bundles supplying the ovules are amphicribral (xylem sandwiched or surrounded by phloem, Fig 2e). Such amphicribral bundle organization is similar to the protostele in early land plants, implying an axial nature for the placenta. Anatomy and morphology seem to agree on that the female units, at least in Magnoliaceae, are composite organs comprising both foliar and axial parts, just as previous works implied [11, 20, 21]. Interestingly, ovules in gymnosperms are also actually borne on branches (not megasporophylls as frequently assumed) [22–24]. Such a Bau-plan shared by diversified female units of angiosperms and gymnosperms units all seed plants together and places a solid foundation for the systematics of seed plants, besides providing a rational provenance for “carpels” in angiosperms.

## Conclusion

Placenta and foliar part in female units of angiosperms are two parts supplied by two vascular bundles of different organizations, derived from two former primordia controlled by two different sets of genes. Placenta is supplied by amphicribral vascular bundles, while the ovarian walls are supplied by collateral bundles. This generalization has been confirmed by various independent studies. These observations prompt a re-evaluating on the nature and provenance of angiosperm “carpels”.
